# Neutropenia induced by Valproic Acid: A case report

**DOI:** 10.1192/j.eurpsy.2022.1889

**Published:** 2022-09-01

**Authors:** N. Baldaquí, G. Anmella, S. Madero, F. Gutierrez, E. Pujal, L. Colomer, A. Giménez-Palomo

**Affiliations:** 1 Hospital Clínic de Barcelona, Bipolar And Depressive Disorders Unit, Institute Of Neuroscience, Barcelona, Spain; 2 Hospital Clínic, Psychiatry, Barcelona, Spain; 3 Hospital Clínic de Barcelona, Institute Of Neuroscience, Barcelona, Spain

**Keywords:** bipolar disorder, neutropenia, valproic acid, adverse effect

## Abstract

**Introduction:**

Valproic acid (VPA) is considered a well-tolerated antiepileptic drug used in Bipolar Disorder as a mood stabilizer. Nevertheless, VPA has been related to several adverse effects. Neutropenia is included as a potential adverse effect, although in clinical practice it is not often measured with regularity.

**Objectives:**

To report a case of a patient with Bipolar Disorder type 2 and Personality Disorder Cluster B treated with VPA with a neutropenia caused by VPA.

**Methods:**

A 61-year-old woman assists to the outpatient psychiatric unit in order to a pharmacological treatment adjustment. A blood test is performed showing a decrease in the levels of neutrophiles in comparison with previous tests. Psychiatric history is revised finding and association between the prescription of VPA and the reduction of neutrophile levels. When this drug was removed, neutrophile levels had increased again up to normal levels.

**Results:**

Due to the relationship between neutropenia and VPA treatment, we decided to discontinue this drug. At the beginning the patient doesn’t agree with the withdrawal of VPA treatment due to its effectiveness in her mood stabilization. Psychoeducation sessions are performed in order to explain risk and benefits of potentials treatment alternatives versus maintaining the same prescription. Finally the patient accepts the switch of the mood stabilizer treatment to oxcarbazepine with a good tolerability and effectiveness.

**Conclusions:**

Periodical blood test monitoring is needed in order to study adverse effects as neutropenia in patients with VPA treatment.

**Disclosure:**

The author has received support from Janssen-Cilag, Otsuka-Lundbeck, Italfármaco, Angelini Pharma and Casen Recordati; and declares no support related to the subject of this article.

